# Deep Learning Based Vehicle Detection and Classification Methodology Using Strain Sensors under Bridge Deck

**DOI:** 10.3390/s20185051

**Published:** 2020-09-05

**Authors:** Rujin Ma, Zhen Zhang, Yiqing Dong, Yue Pan

**Affiliations:** 1 College of Civil Engineering, Tongji University, Siping Road 1239, Shanghai 200092, China; rjma@tongji.edu.cn (R.M.); 1310185@tongji.edu.cn (Z.Z.); 2 College of Electronic and Information Engineering, Tongji University, Caoan Road 4800, Shanghai 201804, China; pan_yue@tongji.edu.cn

**Keywords:** vehicle detection, vehicle classification, strain data, Cascade filtering, artificial neural network, deep learning

## Abstract

Vehicle detection and classification have become important tasks for traffic monitoring, transportation management and pavement evaluation. Nowadays there are sensors to detect and classify the vehicles on road. However, on one hand, most sensors rely on direct contact measurement to detect the vehicles, which have to interrupt the traffic. On the other hand, complex road scenes produce much noise to consider when to process the signals. In this paper, a data-driven methodology for the detection and classification of vehicles using strain data is proposed. The sensors are well arranged under the bridge deck without traffic interruption. Next, a cascade pre-processing method is applied for vehicle detection to eliminate in-situ noise. Then, a neural network model is trained to identify the close-range following vehicles and separate them by Non-Maximum Suppression. Finally, a deep convolutional neural network is designed and trained to identify the vehicle types based on the axle group. The methodology was applied in a long-span bridge. Three strain sensors were installed beneath the bridge deck for a week. High robustness and accuracy were obtained by these algorithms. The methodology proposed in this paper is an adaptive and promising method for vehicle detection and classification under complex noise. It would serve as a supplement to current transportation systems and provide reliable data for management and decision-making.

## 1. Introduction

The detection and classification of vehicles are significant for transportation monitoring and bridge management. The vehicle information is collected for vehicle monitoring and managing, data analysis and visualization and improvement of the transportation system [1]. For example, the vehicle location and weight are essential for the weight-limit inspection. Furthermore, accurate vehicle counts and classification are required for intelligent transportation systems (ITS) and for the performance evaluation of bridge structures.

In the last few decades, various types of sensors have been utilized for vehicle detection, or, furthermore, classification. The most widely used sensors include acoustic sensors, inductive-loop sensors, magnetic sensors, strain sensors and image sensors. These sensors have been studied and applied in traffic infrastructures to monitor passing vehicles. However, several deficiencies still exist in current sensor systems [2,3], including (1) the interruption of traffic during installation, such as inductive-loop sensors, (2) exposure on the road which reduces the durability of sensors, for example, acoustic sensors, (3) subjection to the limited perspective and occlusion problem, for example, image sensors.

Strain sensors, which are often used in bridge engineering for weight measuring systems [4], are inexpensive to install and maintain. They are installed under the bridge deck, thus the traffic will not be interrupted. Furthermore, the sensors are more durable while separated by the deck from wheels and sunlight. Futhermore, by the sensor network design and signal processing, vehicles from different lanes can be separated without occlusion.

In this paper, a strain sensor based methodology for vehicle detection and classification for an orthotropic steel girder (OSG) bridge is proposed. Considering the complex noise from the environment and adjacent lanes, a cascading de-noising method is utilized to detect the vehicle signals. Furthermore, the close-range following vehicles (CRFVs) samples in the extracted samples are identified and processed by a trained artificial neural network (ANN) model and the non-maximum suppression (NMS) algorithm, respectively. In the end, a convolutional neural network (CNN) model is designed and trained to classify the obtained vehicle samples into 11 classes. Research work in this paper provides the following contributions when compared with the previous literature:

- A cascading filter-based vehicle detection method is proposed. It is applied to eliminate the complex noise and extract the vehicle samples from the whole strain signal. Compared with the wavelet transformation method, our method only requires two tuned parameters with low computational cost. Furthermore, it is sensitive to both heavy and light vehicles.
- Training and experiment of a simplified ANN model for CRFVs detection. The machine learning algorithm is capable of identifying the vehicle following signals, which can be vital for vehicle detection in heavy traffic scenes. Furthermore, the following NMS algorithm is effective for separation of CRFVs.
- Provision and evaluation of a residual block-based CNN model for vehicle classification. As an end-to-end method, the deep learning model is produced without any manual intervention, thus is more adaptive than the threshold-based methods and machine learning methods of previous works. The model also reached a 0.980 average precision, meaning both precision and generalization ability were obtained.

The remainder of this paper is organized as follows. Section 2 reviews related works in vehicle detection and classification. Section 3 provides an overview of our methodology. Then, Section 4 and Section 5 introduce the proposed vehicle detection algorithm and vehicle classification method. Meanwhile, the effectiveness of the proposed methodology is validated by an experiment conducted in a real OSG bridge in Section 6. Afterwards, Section 7 analyzes the parameters, states the robustness of our methods and visualizes the weights of the CNN. In Section 8, conclusions are drawn and some suggestions are made for future work.

## 2. Related Works

### 2.1. Sensors for Vehicle Detection

Over the last decades, various sensors have been applied to detect and classify vehicles on road, such as electrical-based, electromagnetic-based, optical-based and so on. Roughly the sensors are divided into embedded and non-embedded types according to their installation locations.

The embedded sensors are installed beneath the road pavement. Among all mainstream embedded sensors, the inductive loop sensors are most widely used for vehicle detection [5]. To obtain the vehicle types, signal features in the frequency domain were extracted by the Fourier Transform for three-class classification [6]. Additionally, the inductive loop signature could also be used in a heavy vehicle tracking scheme [7]. To promote the sensitivity and reduce the cost, polyurethane-based pressure sensors were fabricated. Changes in capacitance due to variation were sensed when vehicles passed over the sensors. The experiment indicated its applicability for axle and speed detection [8]. In addition, improved strain or stress data could be obtained by the particle filled sensors. The sensor sensed passing vehicles after being embedded into the pavement. Signal results show that each peak indicated a passing vehicle [9].

However, the embedded sensors are suffering from traffic interruption at installation or low duration during operation. Thus they are limited in some service scenes.

In recent decades, the non-embedded sensors for vehicle detection have been widely studied. The physical features of moving vehicles are widely considered. Firstly, the sound signals of vehicles were studied. By pre-processing, feature extraction and neurocomputing, the vehicle samples were detected and classified [10]. Considering the features of ferrous metals, the magnetic disturbance from passing vehicles can be detected by magnetic sensors [11]. Furthermore, the configuration and size of a vehicle is quantified by waveform processing, which is described as a magnetic point dipole [12]. Though these sensors are cost-effective and precise in experiments, the environmental noise will affect the precision in complex road scenes.

Moreover, the responses from moving vehicles can also be monitored for detection. For one, the infrared sensors and ultrasonic sensors are active instruments for moving detection. They could be mounted in an overhead configuration, and the speed, length and type were obtained through a combined processing procedure [13]. Furthermore, based on the electrical resistance, the strain sensor senses the vehicles by its deformation. When installed under a bridge deck, the signals of a strain sensor peaks when wheels pass over the observation section [14,15]. Wavelet analysis [16] is proposed to detect a moving vehicle, and furthermore, to recognize the axles as well as classify the vehicle type. However, the signal processing usually requires much parameter tuning works, and sometimes the close-range following vehicles are difficult identifying since the response is similar with a multi-axle vehicle.

Due to the computer vision technology, the image sensor has been applied in vehicle detection. Videos are obtained from the perspective of front or side view [17,18]. Image processing techniques and machine learning methods were used to detect the vehicles in continuous frames. However, the effectiveness of cameras may be unstable in complex traffic condition, such as light changing road, multi-lane road and heavy traffic road. In addition, heavy computation cost is required to realize real-time monitoring.

To conclude, on the one hand, the non-embedded cost-effective sensors are preferred in the case of no traffic interruption and duration. On the other, the highly adaptive and low manual intervention signal processing methods are still required for long-term operation.

### 2.2. Vehicle Classification Methods Based on Machine Learning

Based on the collected vehicle data, classification tasks are often required to assess the vehicle characteristics in transportation engineering. In the last decades, machine learning (ML) based methods [19,20] have served as a powerful tool for data classification. Generally, the collected vehicle data should firstly be annotated by different classes, then the ML model is gradually generated by those optimization algorithms. After the process, the model is capable of predicting the class by inputting a new vehicle sample. There have been researches following the scheme.

Concerning the balance of precision and computation cost, the decision tree had been cross-validated to classify seven radio controlled cars in the laboratory [21]. Nearly 100% accuracy was obtained in the experiment. Furthermore, a vehicle classification system was developed for field road experiments in which a support vector machine (SVM) algorithm was reliable to classify small, medium and combined vehicles [22]. Besides, for axle number-based classification, an artificial neural network (ANN) was designed based on a dataset consisting of 9000 records [23]. After extracting essential features by principal components analysis technique, the model was capable of classifying five types of predetermined vehicles. Moreover, the SVM, k-nearest neighbors (KNN) and ANN algorithms were investigated to recognize different vehicle categories [24], so as to cooperate with the vehicle classification rules of Federal Highway Administration (FHWA).

Though classification results can be obtained by the ML models, these methods may suffer from two difficulties: (1) The in-situ scenes of infrastructures are more complex than the laboratory, noise from the environment and adjacent lanes may cause the algorithms invalid. (2) The algorithms are prone to reach a bottleneck and can not improve even with more data.

Recently, the state-of-the-art deep learning (DL) techniques have been widely applied in classification applications [25]. Among them, the convolutional neural networks (CNNs) have drawn the most attention of researchers. The CNN is capable of extracting local and multi-level features, thus obtaining much higher precision in classification tasks than traditional methods [26]. Mostly the CNN is applied in images which can be regarded as two-dimensional discrete signals. By applying CNN-based object detection methods, the vehicles were segmented from the background with class labels [27,28,29]. When calibrated with real world coordination systems, the spacing information of vehicles was accessed [18]. Combining the image processing-based clustering method, the axles were detected and thus the axle type was obtained [30].

However, because of the limitation of perspective viewing, occlusion of the vehicles is still a challenging problem and may cause the method to become invalid. Besides, the computational cost for real-time image processing is expensive [31], while to the best knowledge of the author, there are few researches on the vehicle detection and classification based on DL using one-dimensional signal data. To inspire the methodology, one-dimensional CNN has already been utilized in damage detection based on vibration data [32].

## 3. Overview of Our Methodology

In this paper, we propose a data-driven methodology, which is a solution for the detection and classification of vehicles using strain data under the bridge deck, containing sensors arrangement, cascade vehicle detection, vehicle following detection and vehicle classification.

**Figure 1:**
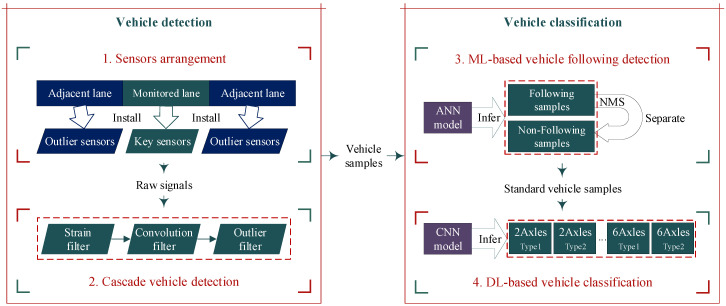
Overview of the proposed methodology.

As indicated in Figure 1, this framework consists of two major parts. In vehicle detection, we propose the arrangement of strain sensors in a bridge section, including key sensors installed under the monitored lane and outlier sensors installed under the adjacent lanes. So the raw time-domain signals are well collected. Then by utilizing three cascading filters (i.e., the strain filter, the convolution filter and the outlier filter), the obtained raw signals are denoised and segmented into vehicle samples in the meantime. In vehicle classification, firstly an ANN model for vehicle following detection is trained and applied to sense the vehicle following samples. By utilizing the NMS algorithm, CRFVs are separated into two vehicles. Thus all standard vehicle samples are obtained. Afterwards, a CNN model for vehicle classification is trained. In the end, the vehicle samples can be inferred as one type from 11 detailed types by the trained CNN model.

## 4. Vehicle Detection by Strain Sensors

Installed under the bridge deck, the strain sensors collect time-domain data, reflecting the local deformation of the deck. When subjected to vehicle axle load, the deck under the wheels will deform itself and a peak occurs in the strain data. Thus the axles are recognized from the peaks and a vehicle is detected from the peak cluster as well.

The wavelet transform is the most commonly used method for peak recognition. After wavelet signal filtering, the peaks are remained and the noise are removed in the signal. The vehicle axles are then detected from the number of the peaks [16]. However, in application, the wavelet transform may not perform well under complex situations. For one, the parameters of the wavelet algorithm require much manual tuning to detect heavy and light vehicles simultaneously. For another, vehicles in adjacent lanes will effect the signals in the monitored lane, which may cause wrong vehicle detection and difficult identification of the tandem-axle and tridem-axle.

In this paper, we propose a cascade method for vehicle detection. Firstly, proper arrangement of sensors is required to collect multi-dimensional strain data for one lane. Secondly, three filters, including strain filter, convolution filter and outlier filter, are applied to eliminate the noise from the environment and from the adjacent lanes. Hence the true axle signals in the specific lane are extracted. Finally, the vehicle samples are obtained by clustering the neighbouring axles.

### 4.1. Sensors Arrangement

**Figure 2:**
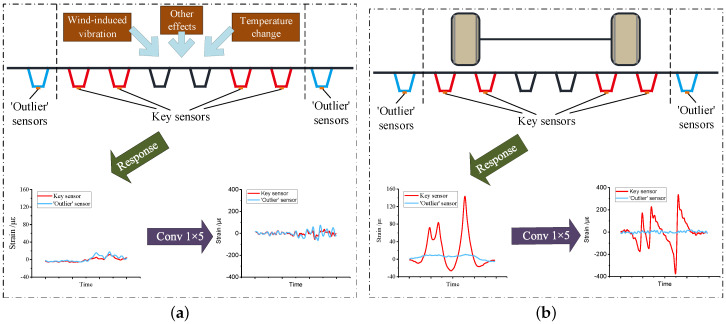

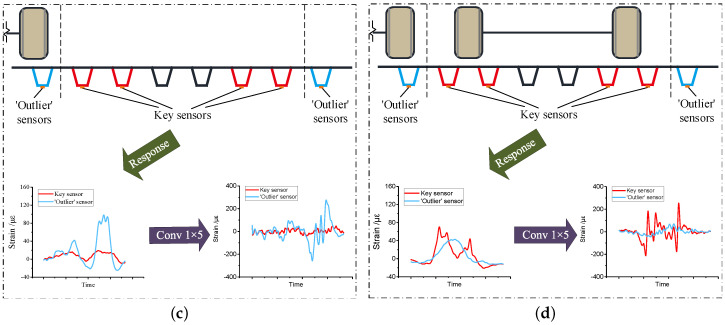
Signal features under different conditions. (**a**) No vehicles crossing the section; (**b**) Vehicle in the monitored lane; (**c**) Vehicle in the adjacent lane; (**d**) Vehicles in both the monitored lane and the adjacent lane.

In order to obtain the strain response, multiple sensors should be installed on the corresponding locations under the deck. On the one hand, sensors installed under the monitored lane are defined as key sensors, which are meant for axle detection. On the the other hand, sensors installed under the adjacent lanes are referred as outlier sensors, which are used to eliminate the noise generated by vehicles on these lanes. The arrangement of the sensors are illustrated in Figure 2. In consideration of the OSG, the key sensors and the outlier sensors would be installed under the U ribs of the monitored lane and the adjacent lanes, respectively.

### 4.2. Signal Features

**Figure 3:**
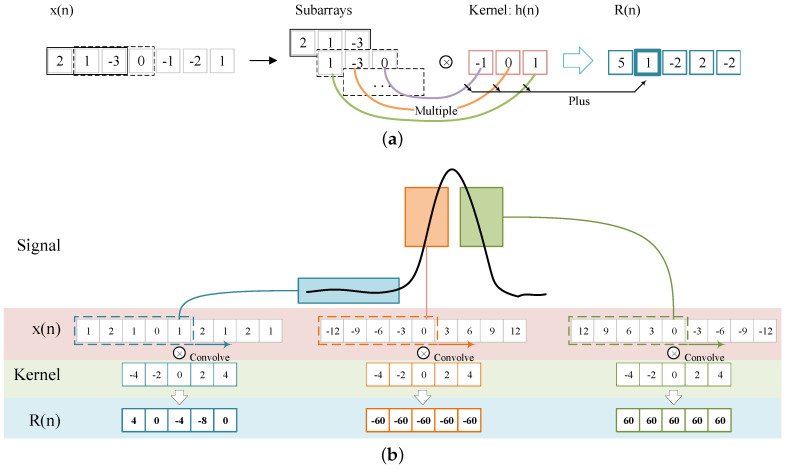
Convolution response of one-dimensional signal. (**a**) One dimensional convolution; (**b**) Convolution response of a vehicle axle.

From the illustration above, peaks are the direct evidence of vehicle axles. Thus, reliable algorithms for the identification of peaks are vital to vehicle detection. As shown in Figure 3b, a pair of rapid increase and rapid decrease is the feature of a peak. The one-dimensional convolution is defined as Equation (1). The x(n) and the h(n) in Equation (1) are two signal series. When the length of h(n) is smaller than x(n), the h(n) degrades as a kernel function, as shown in Figure 3a. The R(n) is denoted as the response of convolution, which is calculated by convolving the subarrays of x(n) and the kernel h(n).

Specifically, three convolution features are found in peak identification. Define a kernel h(n) containing five ascending values (i.e., −4, −2, 0, 2, 4). For the non-peak values, the convolution response is close to zero. Furthermore, for the rapid increase part of a peak, the response will be high positive values. Lastly for the rapid decrease part of a peak, the response will be high negative values. Figure 3b shows an example of the signal convolution.

(1) $$R\left(n\right)=\sum_{i=-\infty }^{+\infty }x\left(i\right)h\left(n-i\right)=x\left(n\right)\times h\left(n\right)$$

The vehicle detection method is designed based on the signal features under different situations. In the field, four possible cases may occur in the test section, causing four types of signal features. As shown in Figure 2, the cases are indicated as follows.

(1) No vehicles in the section, as shown in Figure 2a. The signal only comes from the environment. Hence only slight fluctuation occurs in the strain response. Besides, the convolution responses of both types of sensors are low.
(2) Vehicle in the monitored lane, shown as Figure 2b, meaning the signals mainly reflect the monitored vehicle. There will be several peaks in the key sensor, each referring to a single vehicle axle. Meanwhile, the strain response is much less obvious in the outlier sensor. In addition, much higher convolution response is obtained by the key sensor than the outlier sensor.
(3) Vehicle in the adjacent lane, depicted in Figure 2c. In this case, the outlier sensor draws several peaks when a vehicle crosses. Furthermore, the key sensor will still respond as some fluctuations, meaning the adjacent vehicle causes noise to the monitoring process. In the meantime, the convolution response of a key sensor is lower than the outlier sensor.
(4) Vehicles both in the monitored lane and the adjacent lane, which means the signals will indicate the two vehicles in the meantime. As shown in Figure 2d, the peaks of the strain responses will be close from the key sensor and the outlier sensor. However, strain responses of key sensors will be disturbed and the peaks may not be pure under this condition.

### 4.3. Vehicle Detection

Considering the features of the signal under different conditions, we propose a cascade method for vehicle extraction. From the perspective of Figure 2, three types of noise may interfere with the detection of vehicles, including two from the environment and one from the adjacent lane. Thus, our method consists of three filters to eliminate the non-vehicle signals. After filtering, the principle of proximity is applied to cluster the remaining signal. Hence the vehicle samples are obtained.

#### 4.3.1. Cascade Filtering

The cascade method, proposed in computer science, is an operation to describe the dependency of data or objects [33]. For example, a cascade processing system consists of several methods. When new data are entered into the system, the data will be tested by these methods in an ordered way. In object detection, the boosted cascade based on Haar-like feature was presented and showed robustness in high-level feature extraction [34]. Thus the cascade method is strongly adaptive for selection from multiple conditions.

In this paper, we propose a cascade filtering model containing three filters for vehicle detection. The collected raw strain signal is input into the model and processed by these filters one after the other. The vehicle samples finally remain after the filtering.

**Figure 4:**
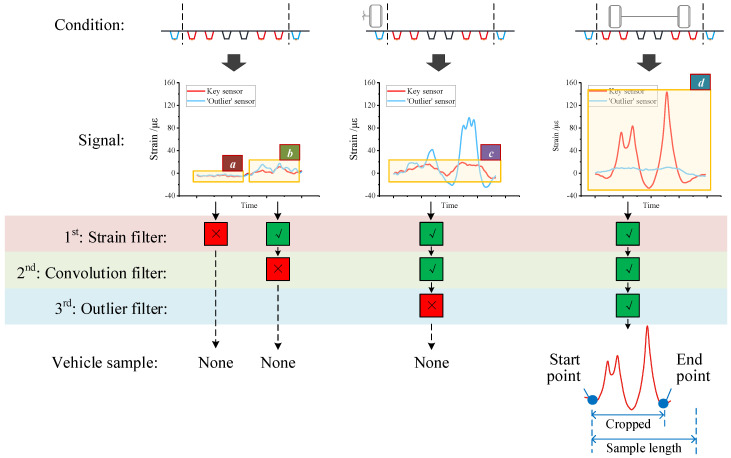
Vehicle detection based on cascade filtering and axle clustering.

Generally, when no vehicles cross the section, sample ⌈a⌋ in Figure 4 features as low strain and low convolution response. In this case, the strain value of sample ⌈a⌋ will not reach the threshold of the filter. So our method removes this type of sample at the first filter (i.e., the strain filter).

Besides, in some environmental cases such as wind-induced vibration, sample ⌈b⌋ in Figure 4 shows high strain response. However, unlike running a concentrated force as vehicle axle, the environmental effect ⌈b⌋ will respond with a low convolution response, as shown in Figure 2a. Our method will remove this type of sample at the second filter (i.e., the convolution filter).

In addition, in case of only vehicles in the adjacent lane, sample ⌈c⌋ in Figure 4 is characterized as higher strain and convolution response than the environmental cases. Hence the first two filters may not recognize this non-vehicle sample. The third filter would be the outlier filter, which compares the convolution response between the key sensor and the outlier sensor. If the response from the key sensor is lower than that from the outlier sensor, the sample will be successfully identified as a non-vehicle sample.

Finally, as for true vehicles in the monitored lane, signal sample ⌈d⌋ in Figure 4 has shown high strain and high convolution response, in both an absolute and relative way. Therefore, the sample will pass through all the proposed three filters, meaning that the vehicle is finally detected. 

#### 4.3.2. Axle Clustering

After the filtering, non-vehicle samples are eliminated from the raw signal, leaving only the peak signal which refers to vehicle axles. To obtain the vehicle samples, the axle clustering method is then applied. The method starts with a high negative convolution response, shown in the bottom-right graph of Figure 4. Then, a sampling window is generated from the start point to cover the whole vehicle signal. Afterward, the method searches for the last positive convolution response and labels the end point. Lastly, the sample signal is precisely cropped from the start point to the end point. Thus, the vehicle samples are obtained with only the vehicle strain response.

## 5. Identification of Vehicle Types

Followed by the detection workflow, vehicle samples are extracted from the raw strain signal. Then the classification of these samples is required. In normal applications, the wavelet transform is used and the peak counting algorithms are applied to identify the axles of vehicles. However, the wavelet is difficult in identifying the CRFV samples because it usually keeps the peak and removes the signal features between peaks. Besides, the wavelet is sensitive to peaks, which are prone to be effected by the axle signal from the adjacent lanes in the OSG bridge.

**Figure 5:**
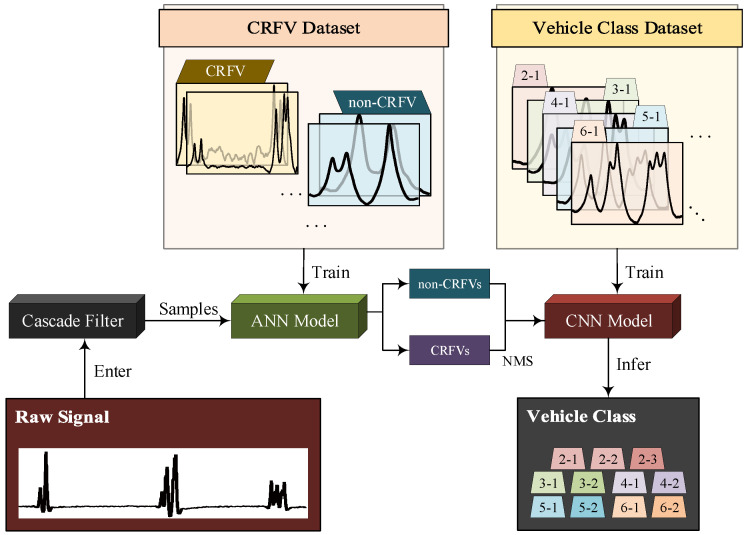
The workflow of vehicle identification method.

Therefore, in this paper, we propose a dual neural network procedure for vehicle samples processing. Firstly, considering the simplicity and time-efficiency of CRFV identification, an ANN model is designed and applied. Furthermore, the identified CRFVs are separated by the NMS method. Finally, after trained and validated, a deep CNN model is utilized to classify the vehicle signal into 11 types. The whole workflow of the proposed vehicle identification method is shown in Figure 5.

### 5.1. Vehicle-Following Identification Based on ANN

Derived from the human neural system, the ANN [35] has always been a powerful tool in machine learning because of its non-linearity, adaptability and high dimensionality. Generally, the ANN is designed as neuron layers transferring information.

**Figure 6:**
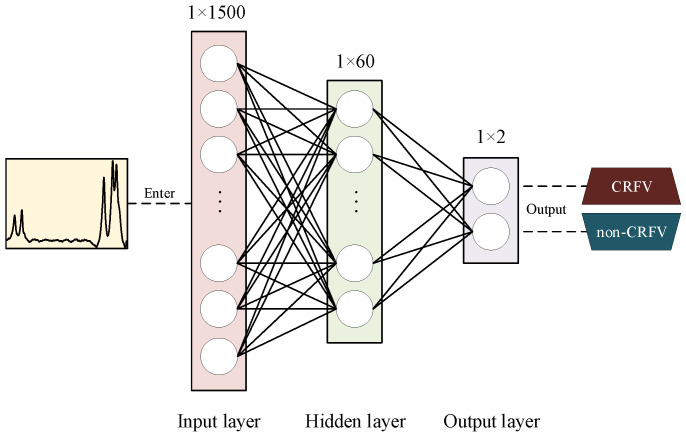
The architecture of the proposed ANN.

In this study, we designed the architecture of the ANN by selecting the number of neurons in each layer. Annotated data with same dimensions are made to train the ANN to distinguish the CRFV samples from the whole detected samples. The structure of the proposed ANN consists of three layers, input layer, hidden layer and output layer. The configuration of layers in this study are designed as follows. Furthermore, the model equation is shown in Equation (2). Architecture of the ANN is depicted in Figure 6.

- The input layer. It consists of 1500 neurons, which matches the length of the input signal. After a full-connected linear weight calculation and a nonlinear ReLU activation function, the results of this layer are transferred to the next layer.
- The hidden layer. It contains 60 neurons, which is optimized by parametric analysis. Linear weight calculation and nonlinear Sigmoid activation function are then applied to obtain the results of this layer.
- The output layer. It is composed of two neurons to represent the probabilities of the two output classes (CRFV and non-CRFV).

(2) $$\left\{\begin{array}{c} t_{j}=f_{R}\left(\sum_{i=1}^{m}\omega_{ij}x_{i}-b_{j}\right),j=1,2,\cdots ,60\\ y_{p}=f_{S}\left(\sum_{k=1}^{n}\nu_{kp}t_{k}-d_{p}\right),p=1,2 \end{array}\right.$$

where *x*, *t* and *y* are the neurons in the input layer, hidden layer and output layer, respectively. ω and ν are the weight parameters, *b* and *d* are the bias parameters between the layers. $f_{R}$ and $f_{S}$ are ReLU function and Sigmoid function, shown in Equation (3).

(3) $$\left\{\begin{array}{c} f_{R}=\max\left(0,x\right)\\ f_{S}=\frac{1}{1+e^{-x}} \end{array}\right.$$

Apart from the architecture, the hyper-parameters for the training are also essential. The Adam [36] method serves best as the optimization function to accelerate the training process. Binary log loss is chosen, which defined as Equation (4), where *N* is the number of training samples, *y* is the code of ground truth (1 for CRFV or 0 for non-CRFV), and *p* is the confidence score to be CRFV computed by the model.

(4) $$L=-\frac{1}{N}\sum_{i=1}^{N}\left(y_{i}\log p_{i}+\left(1-y_{i}\right)\log\left(1-p_{i}\right)\right)$$

### 5.2. CRFV Separation by NMS Method

The NMS algorithm has often been used in image object detection [37] to increase the precision. Concretely, in a local region, the object with the highest confidence is remained. The others are considered to be a bad detection of the same object and is thus deleted.

**Figure 7:**
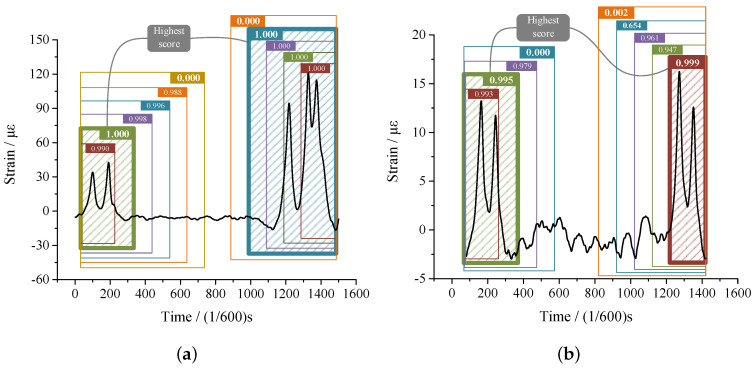
CRFVs separation by NMS method. (**a**) Sample 1; (**b**) Sample 2.

In this study, we apply NMS to the separation of CRFVs. The procedure is presented in Figure 7. Firstly, starting from the beginning of the sample, we select bounding boxes at 100 intervals. Then, the trained ANN model is used to obtain the non-CRFV scores of the boxes. In Figure 7, the scores are shown at the corners of the boxes. Finally, bounding box achieving the highest score is considered the best detection of vehicles. Other boxes with overlapping areas are removed in the meantime. For the second vehicle, the same method with a reverse direction is applied, depicted at the right of Figure 7b clearly.

### 5.3. Vehicle Classification Based on CNN

During the last two decades, Deep Neural Networks (DNN) have been developed rapidly, known as the deep learning [38] technique, which has been widely applied in computer vision (CV), natural language processing (NLP) and speech recognition (SR). For classification tasks, the Convolutional Neural Networks (CNNs) [39,40,41] have shown significant superiority than traditional methods. The convolution module has the ability to obtain local features, which works better than original neural networks for non-uniform data in multi-classification. During last decades, the CNN has been successfully applied in structural health monitoring [42] such as damage detection [43] and signal classification [44].

In this study, the one-dimensional convolution is applied to adapt to the signal processing. As shown in Figure 5, after CRFVs identification, the standard vehicle samples are required to be classified. Thus, a residual block-based CNN is well designed to classify the 11 possible vehicle types.

#### 5.3.1. Types and Strain Signals of Axle Group

**Figure 8:**
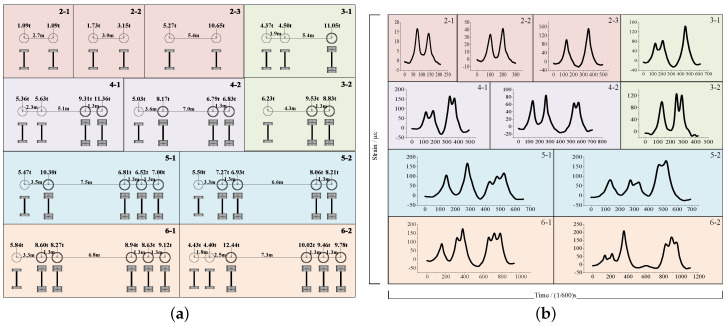
Types of axle group for the studied transportation system. (**a**) Statistical features; (**b**) Strain signal features.

In this study, 11 classical types of axle group are collected from our previous research. The statistical and strain features of these types are shown in Figure 8. The vehicles are coded based on their axle number, axle distribution and axle load.

#### 5.3.2. CNN Model

As a key operation in CNN, the convolution operation is defined in Equation (1). Contrary to the vehicle detection scheme, values of the convolution kernel in CNN will be self-learned in the model training. Basically, the CNN is composed of a convolution layer and pooling layer. In the convolution operation, local features are extracted with deeper dimensions. After convolution, the pooling layer follows which reduces the feature size, which provides extractions for deeper features.

**Figure 9:**
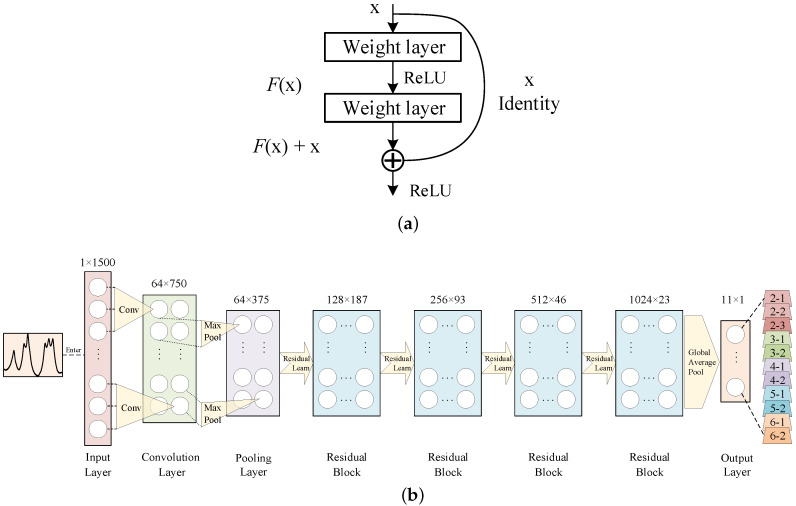
The architecture of the proposed ANN and CNN model. (**a**) Building block of residual learning; (**b**) The architecture of the proposed ResNet model.

In consideration of the deep feature extraction, the residual learning block [40] is used in our CNN. As illustrated in Figure 9b, rather than regular pass-through connections, the residual block applies a shortcut method that connects the input to the output. Thus, when the neural network goes deeper, the residual mapping works to avoid the gradient disappearance problem. In other words, in the training process, the residual neural network optimize parameters by reducing the residual errors rather than the original errors. The block is defined as Equation (5).

(5) $$y=F(x,\left\{W_{i}\right\})+x$$

Here, *x* and *y* are the input and output vectors of the layers. *F* is the residual mapping to be learned and $\left\{W_{i}\right\}$ are the weights.

By connecting the input signal, convolutional layer, pooling layer, four residual blocks and global average pooling, the CNN for vehicle classification is built. The whole architecture of the CNN is depicted in Figure 9c.

For the training of the CNN, we select and tune the hyper-parameters. The Adam optimizer is applied in the training for its high efficiency and high adaptability. Besides, the multi cross-entropy log loss is chosen to compute the distance between predicted labels and true labels. The L2-regularization is used to reduce the overfitting. The combined loss function is defined in Equation (6). Where *t* represents the class label. λ is the punishment index for regularization. Furthermore, $W^{l}$ is the weight matrix of layer *k*.

(6) $$L=-\frac{1}{N}\sum_{i=1}^{N}\left(y_{i}^{\left(t\right)}\log p_{i}^{\left(t\right)}+\left(1-y_{i}^{\left(t\right)}\right)\log\left(1-p_{i}^{\left(t\right)}\right)\right)+\frac{1}{N}\frac{\lambda }{2}\sum_{k}|W_{k}{|}^{2}$$

## 6. Case Study

In this section, the proposed methodology was applied and evaluated in the steel box girder of a 15-year suspension bridge. Firstly, strain sensors were arranged and installed under the U-ribs of the bridge deck. Strain-time data of the outermost lane were collected over seven days. Then, through the proposed filters, vehicle samples were extracted from the raw strain signals. Furthermore, with the assistance of ANN model and NMS method, the CRFVs were separated effectively. Finally, a CNN model for signal classification was trained and evaluated on a dataset with 7295 samples, which were manually annotated from field experiment. The model is practical for multi-class vehicle identification.

### 6.1. General Information

The experiment was carried out in the steel box girder of a 15-year suspension bridge. The main span of the bridge was 1490 m. The width and height of the girder were 38.7 and 3.0 m, respectively. The bridge contained a total of six lanes, each with a 4-meter width.

**Figure 10:**
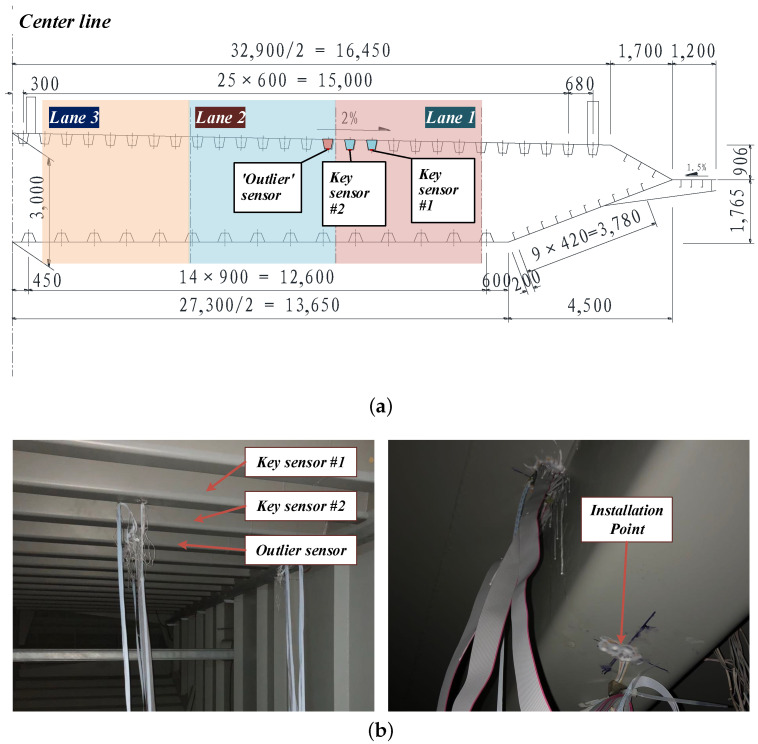
The sensor arrangement in the experiment. (**a**) The section drawing and sensor positions; (**b**) The in-situ installation of the strain sensors.

The strain sensors were installed as Figure 10 shown. The signal acquisition channels were synchronized with each other. The parameters were listed in Table 1. It is worth mentioning that the frequency of the sensors was set to 600 because in low acquisition frequency, response of low-weight vehicles are too small to be identified. For clarity, the sensor under the 11th U-rib (i.e., the key sensor #1) served as the key sensor and the following signal analysis was based on its data.

The PyTorch framework [45] was used to perform all the experiments and studies in this article. To implement the training and evaluation of neural networks, a desktop PC (CPU: IntelR CoreTM i7-6700k; RAM: 32 GB and GPU: NVIDIA Geforce GTX 1080Ti) with the support of CUDA v10.0 and cuDNN v7.4 was used.

### 6.2. Vehicle Detection

#### 6.2.1. Vehicle Extraction by Cascade Filtering

**Figure 11:**
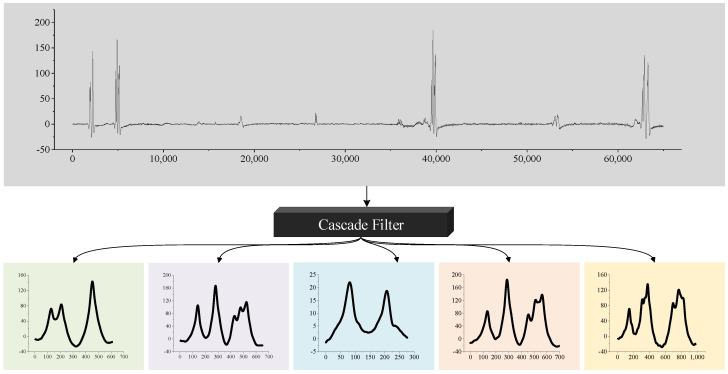
Vehicle samples extraction by the proposed cascade filter.

First of all, the cascade filter was set by setting parameters for strain filter, convolution filter and outlier filter. Among them, the thresholds for strain and convolution filter were 5 and 25, respectively. Signals and their convolution response under the thresholds were removed and considered not vehicles. Besides, the outlier filter was applied to remove the signals when the convolution response of outlier sensor is greater than the key sensor’s. After all the filtering, vehicle samples were obtained, as shown in Figure 11. Finally, 29,915 vehicle samples were collected by applying the method to the 7-day strain data.

#### 6.2.2. ANN Model Training and Evaluation

After cascade filtering, model for CRFVs identification is required. In this study, we manually annotate a dataset, including 570 CRFV samples and 660 non-CRFV samples for ANN training. By random selection, the dataset is divided into three groups: 738 samples for training, 246 samples for validation and the rest 246 samples for test.

The main hyper-parameters of ANN training are listed in Table 2. The loss function and optimizer are 2D Cross-Entropy and Adam [36], respectively. The learning rate (LR) will be reduced by 0.5 every 5 epochs.

**Figure 12:**
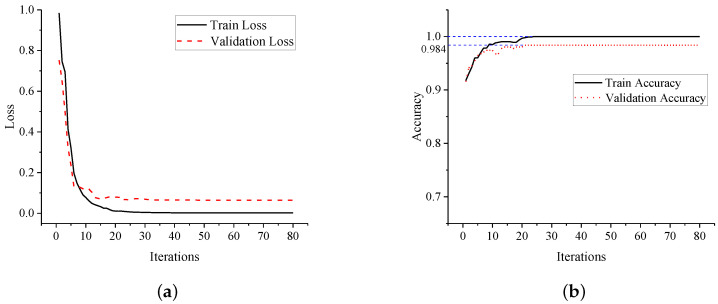
The training curves of the proposed ANN model. (**a**) Loss curve of the ANN model; (**b**) Accuracy curve of the ANN model.

In order to monitor the training process, the loss curve and the accuracy curve of dataset are illustrated in Figure 12. Obviously, the loss is decreasing with iterations, which means the errors are decreasing and the parameters are optimized gradually. Finally, the model converges at about its 25th iteration, when the loss value is approaching 0 and the accuracy value is approaching 1.

From Figure 12b, the proposed ANN model manages to handle the under-fitting and over-fitting problem. The final accuracy of the training set and validation set are 1.000 and 0.984, respectively. After training, the optimized ANN model is obtained. By applying the model to detect CRFV samples in the test set, a precision of 0.986 is finally achieved. Besides, the confusion matrix of the test set is shown in Table 3.

#### 6.2.3. CRFV Identification and Separation

**Figure 13:**
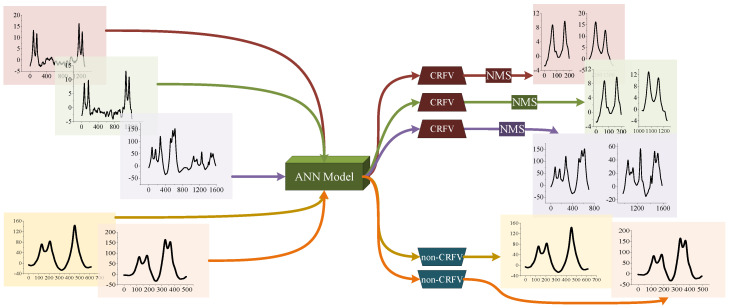
Examples of CRFV identification and separation.

After it is trained, the ANN model is applied to the obtained vehicle samples to identify CRFVs. In the experiment, A total of 946 CRFV samples were found; examples are shown in Figure 13. Obviously, because of the high speed, two-axle vehicles tended to occur in the CRFVs.

### 6.3. Vehicle Classification

#### 6.3.1. Dataset Generation

A dataset is essential to the deep neural network. To obtain a high precision model, we manually annotated a large dataset, including 7295 vehicle strain signal samples. The components of the dataset are listed in Table 4. Because the key sensor was installed under the outermost lane to mainly capture the heavy vehicles, six-axle vehicles are in a majority in the dataset. The whole dataset was then divided into three parts by 0.6, 0.2, and 0.2; that is, 4377, 1459, and 1459 samples for training, validation and test, respectively.

#### 6.3.2. CNN Model Training

**Figure 14:**
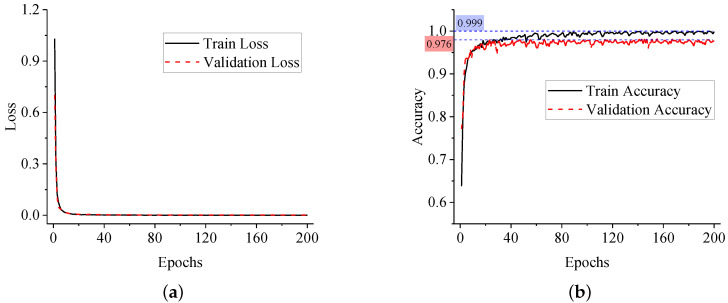
The training curves of the proposed CNN model. (**a**) Loss curve of the CNN model; (**b**) Accuracy curve of the CNN model.

The training process of the proposed CNN model is monitored by plotting the loss and accuracy curve, as shown in Figure 14. From the curves, the loss was continuously decreasing throughout the training. The convergence point was found at about 20 epochs, which meant the model was easily trained with little under-fitting. Finally the accuracy of the training set and validation set were reached at 0.999 and 0.976.

#### 6.3.3. CNN Model Evaluation

**Figure 15:**
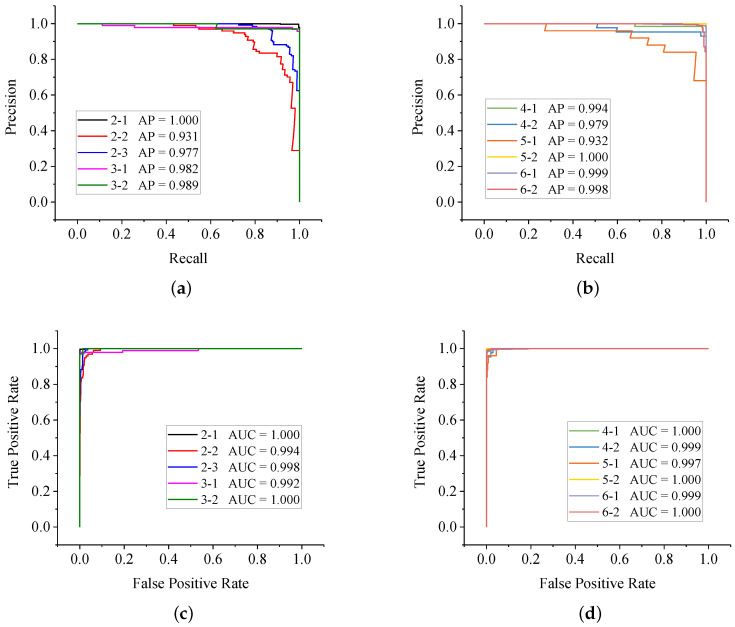
The evaluation curves of the proposed ResNet model. (**a**) PR curve of the ResNet model (part 1); (**b**) PR curve of the ResNet model (part 2); (**c**) ROC curve of the ResNet model (part 1); (**d**) ROC curve of the ResNet model (part 2).

After being trained, inference on the test set was carried out to evaluate the generalization ability of the model. In this study, the PR curve and the ROC curve are displayed in Figure 15. For clarity, curves of the 11 classes were divided into part 1 and part 2.

From the PR curve, average precision (AP) of each class was computed. The model behaved outstandingly in identify 2-1, 4-1, 5-2, 6-1 and 6-2. Almost 1.0 AP was obtained in these tasks. However, the model behaved less well for 2-2 and 5-1 because the imbalance of the dataset caused worse performance for the classes with fewer samples. Besides, from the ROC curve, the area under curve (AUC) of each class was obtained. Similar conclusions could be drawn like the PR curve. Eventually, by averaging the APs of each class, the mean average precision (mAP) of the model is 0.980. The confusion matrix could also be obtained by setting thresholds from 0 to 1, shown in Table 5.

## 7. Discussion

In this section, some issues are proposed and discussed. Firstly, the thresholds of the proposed cascade filter are analyzed, in which two indices are defined to obtain the optimum values. Then, part of weight parameters of the CNN model are visualized, from which some patterns are found in the signal classification. Besides, by correctly identifying some noised samples in the test set, the robustness of the proposed CNN model is evaluated. Furthermore, some state-of-the-art architectures of CNN are applied to the vehicle classification. Results of the mAP and inference time of these models are obtained. Finally, WIM data of the same lane during the experiment are analyzed. Compared to the vehicles identified by the proposed method, the effectiveness and deviation are analyzed.

### 7.1. Thresholds Analysis of Cascade filter

**Figure 16:**
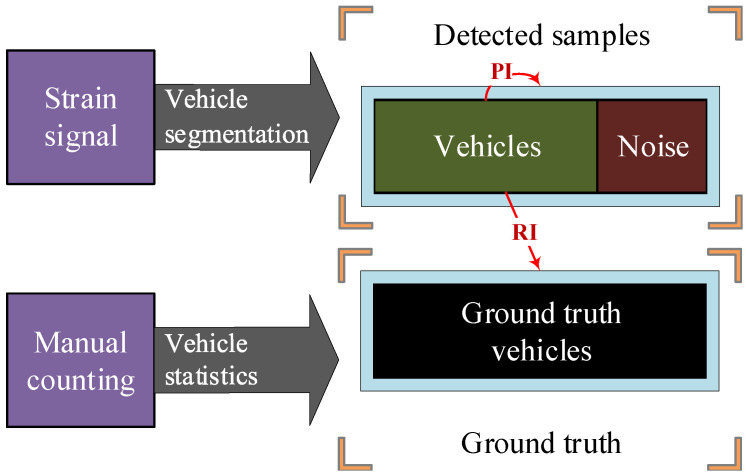
The definition of PI and RI.

In vehicle detection, we use strain, convolution and outlier filters for the cascade filtering. Among them, two thresholds $T_{strain}$ and $T_{conv}$ should be firstly determined. Different combination of the thresholds will effect the detection results. Hence a parametric experiment was carried out. We firstly manually counted vehicles over two hours. Then the corresponding signal was analyzed in different thresholds. To quantitatively evaluate the effects after filtering, two indicators, i.e., Recall Index (RI) and Precision Index (PI), are defined in Figure 16. On the one hand, the PI is the ratio of detected vehicles to the total detected samples. It indicates how correctly the algorithm could distinguish vehicles against noises. On the other hand, the RI is the ratio of detected vehicles to the actual vehicles. It describes how completely the algorithm could search all the vehicles.

**Figure 17:**
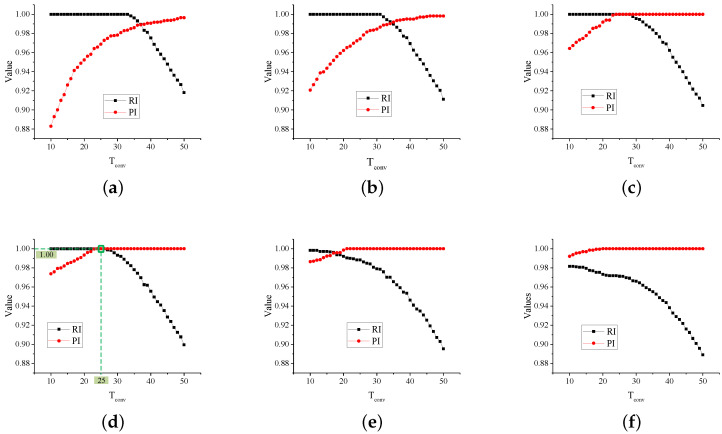
The parametric analysis of thresholds in cascade filtering. (**a**) $T_{strain}=2$; (**b**) $T_{strain}=3$; (**c**) $T_{strain}=4$; (**d**) $T_{strain}=5$; (**e**) $T_{strain}=6$; (**f**) $T_{strain}=7$.

Some patterns are found in figures from (a) to (f) in Figure 17. For one, with $T_{strain}$ increase, the RI of the algorithm tends to decrease because large strain thresholds prevent detection for some light-weight two-axle vehicles. On the contrary, the PI will increase considering the noise will be more effectively intercepted. For another, with $T_{conv}$ increase, the RI tends to decrease because low-speed vehicles may not be detected for its low convolution response. In contrast, the PI will increase in consideration of the high requirement of the convolution response in avoiding detection of vehicles from adjacent lanes.

Thus, thresholds reaching 1.0 in PI and RI are preferred to applied in the vehicle detection algorithm. In this study, the $T_{strain}$ and $T_{conv}$ are determined as 5 and 25, respectively, as shown in Figure 17d.

### 7.2. Visualize of the CNN

**Figure 18:**
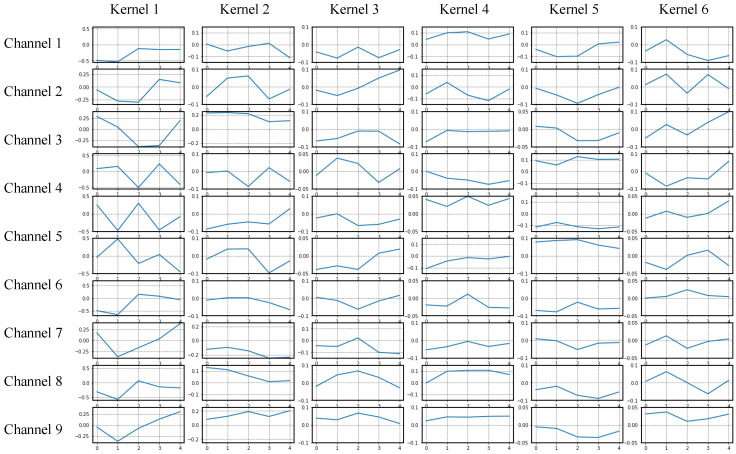
Visualization of kernels for the trained CNN.

In the architecture of CNN, kernels are the main parameters of the convolution layer. They are randomly initialized at the beginning, optimized gradually in the training process. To understand the feature extraction scheme, we generated the window functions in each channel of the first six convolution kernels in the trained CNN, shown in Figure 18.

Clearly, the convolution kernels have learned the strain features of vehicles. Unlike the kernel in vehicle detection, the learned values of kernel were about −0.5 to 0.5 in various frequency. Furthermore, the values tended to decrease with layers going deeper, which may explain why higher features were extracted by deeper kernels. Besides, different from traditional methods, parameters of the signal processing of CNN are all automatically trained by the annotated vehicle data. Thus the CNN not only requires no manual design of signal processing filters, but also more easily adapts to the field application because the model learns feature patterns from in-situ data.

### 7.3. Robustness of the CNN

**Figure 19:**
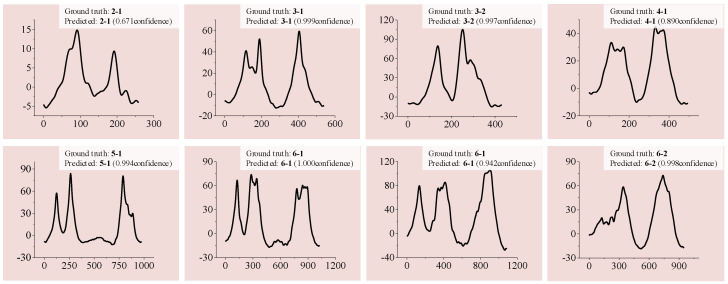
Examples of classification for noised signals.

In some situations, noise can be laborious to describe mathematically. Thus it is difficult for traditional algorithms to adapt to the in-situ multi-class vehicle classification. However, in our experiment, the trained CNN is capable of classifying the examples with little noise, which shows its adaptability for field application. Some examples are depicted in Figure 19. Isolated small peaks may occur because adjacent vehicles were crossing the section at the same time.

Therefore, the robustness and parameter-adaptive of our method are validated. Furthermore, the detailed mathematical procedures can be saved effectively.

### 7.4. Vehicle Classification Comparing with Previous Works

In this study, the deep learning-based method was applied to classify 11 different types of vehicles at 0.980 average precision. Compared with some previous works for vehicle classification, the method performs better both in multi-classification and higher precision, as depicted in Table 6. Our method shows advantages in the highly multi-class vehicle classification and the average precision.

### 7.5. Vehicle Classification by Different CNN Architectures

**Figure 20:**
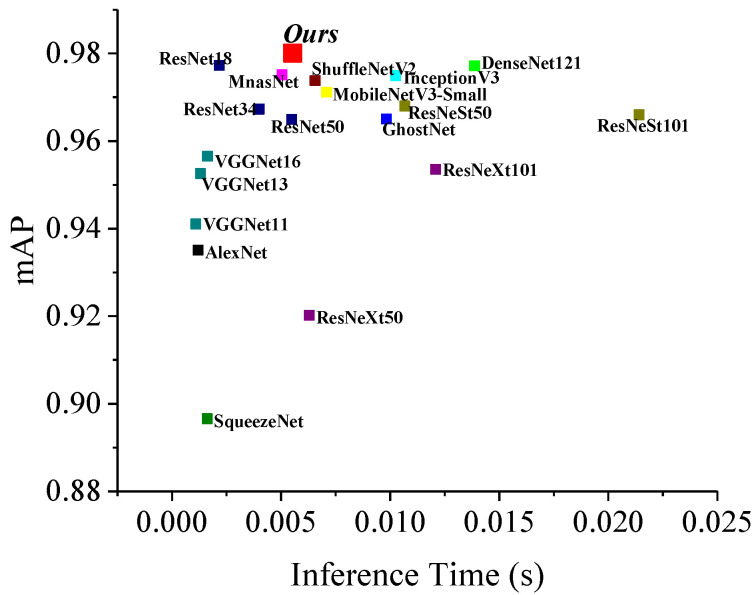
mAP and inference time for vehicle classification by the studied CN.

In last decades, numbers of CNN architectures were proposed and evaluated by researchers, some have been widely used in computer vision filed, such as AlexNet [46], VGGNet [47], Inception [39], ResNeXt [48], ResNeSt [49], MobileNet [50], DenseNet [51], ShuffleNet [52], MnasNet [53], DarkNet [54] and GhostNet [55]. Two indices, mAP and inference time, represent precision and processing time for vehicle classification, respectively. Thus, by training, the CNNs were evaluated and the results are listed in Figure 20. Though the inference times of our model are not the best, the mAP of it is outstanding.

### 7.6. Comparison with WIM Data

A weight-in-motion (WIM) system was previously installed near the experiment section (∼30 m). As a comparative study, the seven-day WIM data of Lane 1 were statistically counted by the vehicle types, listed in Table 7. In total, 30,246 vehicles were collected by the WIM system, which was 1.09% more than the vehicles from our method. In detail, maximum errors were in type 2-1 and 2-2, approximately 5%. Yet the errors of type 6-1 and 6-2 were only 0.23% and 0.26%, which validated the precision of the proposed method.

The deviation of the detected vehicles comes from two aspects. Firstly, deviation from possible lane changing. The distance between the WIM section and the experimented section is 30 m, which means vehicles may change lanes during the interval. Furthermore, it explains why the number of detected vehicles and WIM vehicles are not equal. More testing sections may handle the problem in future research. Secondly, deviation from the cascading thresholds. Reconciling the demands of all vehicles, light or weight, is difficult, though the proposed cascade filtering managed to reach 100% both in precision and recall. Severe noise may be unable to be eliminated and may be identified as vehicles, and some unloaded vehicles may be omitted because of the strain filter. Thirdly, deviation from the ANN model and the CNN model. From the confusion matrices of ANN and CNN, the models may misjudge some uncommon types of vehicles in a 2–3% possibility. By optimizing the hyper-parameters and the architectures of the neural network models, the classification errors can be further reduced.

## 8. Conclusions

A deep learning based methodology for vehicle detection and classification is proposed in this study. The strain sensors are firstly arranged, including the key sensors and outlier sensors for the monitored lane and adjacent lanes. In consideration of the possible loading conditions, signal features are analyzed on its peak features and convolution features. In order to detect vehicles from the strain signal, we then propose a cascading filter consisting of strain filter, convolution filter and outlier filter. The strain and convolution filters are designed to eliminate environmental noises, and the outlier filter is to remove the noise from adjacent vehicles. After an axle clustering scheme, the vehicle samples can be obtained. Afterward, an ANN is introduced to identify the CRFVs in the original vehicle samples. The ANN model is fine trained to 0.986 accuracy on the test set, meaning it is highly reliable for CRFVs recognition. An NMS algorithm is followed to process the CRFVs into two separated vehicles. All the standard vehicle samples are thus obtained after these procedures. Lastly, we design and train a deep learning-based CNN model from a manually annotated dataset. The CNN model is optimized and eventually the mAP reached 0.980. Among all the types, the AP of 2-1 and 6-1 have been obtained as 1.000 and 0.999, respectively.

The methods are all applied in a real OSG bridge to monitor vehicles in the first lane for seven days. The detection and classification of vehicles are experimented and evaluated, which indicates the deep learning-based methodology proposed in this study is feasible for the tasks with high precision.

At the discussion part, we analyze the thresholds of the cascading filter, visualize the parameters of the CNN model, evaluate the CNN model in noised samples, compare our results with other architectures of CNN and analyze the possible deviation by WIM data. In summary, the proposed methodology is effective in the vehicle detection and classification using strain data. With no requirement of traffic interruption, the methodology also has shown strong flexibility, portability and high accuracy. It will be helpful for transportation management and monitoring.

In the future, more complex conditions will be studied, such as traffic jams, lane-changing and variation of the volume of traffic. Furthermore, the thresholds can be designed as self-adjustable to adapt to different scenes. Besides, the effects of the ANN model and CNN model used in this work will be further explored, including the extension of the dataset, fine-tuned hyper-parameters, and so on. 

## Figures and Tables

**Table 1:** Parameters of the applied strain sensors.

Parameters	Units	Values
Frequency	Hz	600
Precision	μϵ	1

**Table 2:** The main hyper-parameters of the proposed ANN and CNN models.

Architecture	Loss Function	Optimizer	Initial LR	Epochs
ANN	2D Cross-Entropy	Adam	0.001	80
CNN: ResNet	Multi Cross-Entropy	Adam	0.001	200

**Table 3:** Confusion matrix of the proposed ANN model.

		Predict	
		**CRFV**	**Non-CRFV**
True	CRFV	140	4
	non-CRFV	1	163

**Table 4:** Vehicle number of different types in our dataset.

Type	2-1	2-2	2-3	3-1	3-2	4-1	4-2	5-1	5-2	6-1	6-2	Total
Number	1427	484	642	467	170	311	216	122	40	2334	1082	7295

**Table 5:** Confusion matrix of the ResNet model.

		Predict										
		**2-1**	**2-2**	**2-3**	**3-1**	**3-2**	**4-1**	**4-2**	**5-1**	**5-2**	**6-1**	**6-2**
True	2-1	290	3	0	0	0	0	0	0	0	0	0
	2-2	1	90	9	0	0	0	0	0	0	0	0
	2-3	0	11	117	1	0	0	0	0	0	0	0
	3-1	0	0	2	96	0	0	0	0	0	0	0
	3-2	1	2	0	1	33	0	0	0	0	0	0
	4-1	0	0	0	1	0	65	0	0	0	0	0
	4-2	0	0	0	0	0	0	45	0	0	0	0
	5-1	0	0	0	0	0	0	2	20	0	0	1
	5-2	0	0	0	0	0	0	1	0	6	0	1
	6-1	0	0	0	0	0	0	1	2	0	473	0
	6-2	0	0	0	0	0	1	0	2	2	0	216

**Table 6:** Vehicle classification comparing with previous works.

No.	Sensor	Classification Method	Number of Classes	Precision	In-Situ Environment
1 [6]	Inductive loop	FT	3	96.44%	Yes
2 [10]	Acoustic	ANN	4	86.00%	Yes
3 [13]	Infrared/Ultrasonic	BN	5	84.74%	Yes
4 [18]	Vision	DNN	8	81.50%	Yes
5 [22]	Fiber	SVM	3	98.50%	Yes
6 [21]	Magnetometer	DT	7	97.62%	No
7 [23]	Strain	ANN	5	95.38%	Yes
8 (ours)	Strain	DNN	11	98.01%	Yes

**Table 7:** Comparison of detected vehicle between our method and WIM system.

Type	2-1	2-2	2-3	3-1	3-2	4-1	4-2	5-1	5-2	6-1	6-2	Total
Count (Ours)	4953	2850	2567	2181	364	1320	1019	716	226	9491	4228	29,915
Count (WIM)	5195	3012	2503	2163	352	1296	1042	742	233	9469	4239	30,246
Error (%)	4.66%	5.38%	2.56%	0.83%	3.41%	1.85%	2.21%	3.50%	3.00%	0.23%	0.26%	1.09%

## References

[1] Gupte S., Masoud O., Martin R.F.K., Papanikolopoulos N.P. (2002). Detection and classification of vehicles. IEEE Trans. Intell. Transp. Syst..

[2] Mimbela L.E., Klein L., Luces K. Summary of Vehicle Detection and Surveillance Technologies Used in Intelligent Transportation Systems. https://www.fhwa.dot.gov/policyinformation/pubs/vdstits2007/vdstits2007.pdf.

[3] Ahmed W., Arafat S.Y., Gul N. A Systematic Review on Vehicle Identification and Classification Techniques. Proceedings of the 2018 IEEE 21st International Multi-Topic Conference (INMIC).

[4] Yu Y., Cai C.S., Deng L. (2016). State-of-the-art review on bridge weigh-in-motion technology. Adv. Struct. Eng..

[5] Gajda J., Piwowar P., Sroka R., Stencel M., Zeglen T. (2012). Application of inductive loops as wheel detectors. Transp. Res. Part C Emerg. Technol..

[6] Lamas-Seco J., Castro P., Dapena A., Vazquez-Araujo F. (2015). Vehicle Classification Using the Discrete Fourier Transform with Traffic Inductive Sensors. Sensors.

[7] Jeng S.T., Chu L. (2015). Tracking Heavy Vehicles Based on Weigh-In-Motion and Inductive Loop Signature Technologies. IEEE Trans. Intell. Transp. Syst..

[8] Chien K.D., Van-Phuc H., Duy T.N., Toan T.D. (2019). A Low-Cost, Flexible Pressure Capacitor Sensor Using Polyurethane for Wireless Vehicle Detection. Polymers.

[9] Han B., Zhang K., Yu X., Kwon E., Ou J. (2011). Nickel particle-based self-sensing pavement for vehicle detection. Measurement.

[10] Nooralahiyan A.Y., Kirby H.R., McKeown D. (1998). Vehicle classification by acoustic signature. Math. Comput. Model..

[11] Sifuentes E., Casas O., Pallas-Areny R. (2011). Wireless Magnetic Sensor Node for Vehicle Detection with Optical Wake-Up. IEEE Sens. J..

[12] Zhu H., Yu F. (2016). A Cross-Correlation Technique for Vehicle Detections in Wireless Magnetic Sensor Network. IEEE Sens. J..

[13] Odat E., Shamma J.S., Claudel C. (2018). Vehicle Classification and Speed Estimation Using Combined Passive Infrared/Ultrasonic Sensors. IEEE Trans. Intell. Transp. Syst..

[14] Yu Y., Cai C.S., Deng L. (2015). Vehicle axle identification using wavelet analysis of bridge global responses. J. Vib. Control.

[15] Kalhori H., Makki A.M., Zhu X., Samali B. (2018). Nothing-on-Road Axle Detection Strategies in Bridge-Weigh-in-Motion for a Cable-Stayed Bridge: Case Study. J. Bridge Eng..

[16] Chatterjee P., OBrien E., Li Y., Gonzalez A. (2006). Wavelet domain analysis for identification of vehicle axles from bridge measurements. Comput. Struct..

[17] Sang J., Wu Z., Guo P., Hu H., Xiang H., Zhang Q., Cai B. (2018). An Improved YOLOv2 for Vehicle Detection. Sensors.

[18] Zhang B., Zhou L., Zhang J. (2018). A methodology for obtaining spatiotemporal information of the vehicles on bridges based on computer vision. Comput. Aided Civ. Infrastruct. Eng..

[19] Cortes C., Vapnik V. (1995). Support-Vector Networks. Mach. Learn..

[20] Breiman L. (2001). Random forests. Mach. Learn..

[21] Ying K., Ameri A., Trivedi A., Ravindra D., Patel D., Mozumdar M. Decision Tree-based Machine Learning Algorithm for In-node Vehicle Classification. Proceedings of the IEEE Green Energy and Systems Conference (IGESC).

[22] Al-Tarawneh M., Huang Y., Lu P., Tolliver D. (2018). Vehicle Classification System Using In-Pavement Fiber Bragg Grating Sensors. IEEE Sens. J..

[23] Yan L., Fraser M., Elgamal A., Fountain T., Oliver K. (2008). Neural networks and principal components analysis for strain-based vehicle classification. J. Comput. Civ. Eng..

[24] Al-Tarawneh M., Huang Y. Road vehicle classification using machine learning techniques. Proceedings of the SPIE Smart Structures + Nondestructive Evaluation.

[25] Schmidhuber J. (2015). Deep learning in neural networks: An overview. Neural Netw..

[26] Gu J., Wang Z., Kuen J., Ma L., Shahroudy A., Shuai B., Liu T., Wang X., Wang G., Cai J. (2018). Recent advances in convolutional neural networks. Pattern Recognit..

[27] Dan D., Ge L., Yan X. (2019). Identification of moving loads based on the information fusion of weigh-in-motion system and multiple camera machine vision. Measurement.

[28] Gomaa A., Abdelwahab M.M., Abo-Zahhad M., Minematsu T., Taniguchi R.i. (2019). Robust Vehicle Detection and Counting Algorithm Employing a Convolution Neural Network and Optical Flow. Sensors.

[29] Wu Z., Sang J., Zhang Q., Xiang H., Cai B., Xia X. (2019). Multi-Scale Vehicle Detection for Foreground-Background Class Imbalance with Improved YOLOv2. Sensors.

[30] Yao Z., Wei H., Li Z., Corey J. (2016). Fuzzy C-Means Image Segmentation Approach for Axle-Based Vehicle Classification. Transp. Res. Rec..

[31] Chen Y., Hu W. (2020). Robust Vehicle Detection and Counting Algorithm Adapted to Complex Traffic Environments with Sudden Illumination Changes and Shadows. Sensors.

[32] Zhang Y., Miyamori Y., Mikami S., Saito T. (2019). Vibration-based structural state identification by a 1-dimensional convolutional neural network. Comput. Aided Civ. Infrastruct. Eng..

[33] Motter A.E., Lai Y.C. (2002). Cascade-based attacks on complex networks. Phys. Rev. E.

[34] Lienhart R., Maydt J. An extended set of haar-like features for rapid object detection. Proceedings of the IEEE International Conference on Image Processing ICIP.

[35] Rumelhart D.E., Hinton G.E., Williams R.J. (1986). Learning representations by back-propagating errors. Nature.

[36] Kingma D., Ba J. (2014). Adam: A Method for Stochastic Optimization. arXiv.

[37] Ren S., He K., Girshick R., Sun J. (2017). Faster R-CNN: Towards Real-Time Object Detection with Region Proposal Networks. IEEE Trans. Pattern Anal. Mach. Intell..

[38] LeCun Y., Bengio Y., Hinton G. (2015). Deep learning. Nature.

[39] Szegedy C., Liu W., Jia Y., Sermanet P., Reed S., Anguelov D., Erhan D., Vanhoucke V., Rabinovich A. Going Deeper with Convolutions. Proceedings of the 2015 IEEE Conference on Computer Vision and Pattern Recognition (CVPR).

[40] He K., Zhang X., Ren S., Sun J. Deep Residual Learning for Image Recognition. Proceedings of the 2016 IEEE Conference on Computer Vision and Pattern Recognition (CVPR).

[41] Howard A.G., Zhu M., Chen B., Kalenichenko D., Wang W., Weyand T., Andreetto M., Adam H. (2017). MobileNets: Efficient Convolutional Neural Networks for Mobile Vision Applications. arXiv.

[42] Dong C.Z., Catbas N. (2020). A review of computer vision–based structural health monitoring at local and global levels. Struct. Health Monit..

[43] Cha Y.J., Choi W., Suh G., Mahmoudkhani S., Büyüköztürk O. (2018). Autonomous Structural Visual Inspection Using Region-Based Deep Learning for Detecting Multiple Damage Types. Comput. Aided Civ. Infrastruct. Eng..

[44] Oh B.K., Glisic B., Kim Y., Park H.S. (2019). Convolutional neural network-based wind-induced response estimation model for tall buildings. Comput. Aided Civ. Infrastruct. Eng..

[45] Paszke A., Gross S., Massa F., Lerer A., Bradbury J., Chanan G., Killeen T., Lin Z., Gimelshein N., Antiga L. (2019). PyTorch: An Imperative Style, High-Performance Deep Learning Library. arXiv.

[46] Krizhevsky A., Sutskever I., Hinton G. (2012). ImageNet Classification with Deep Convolutional Neural Networks. Neural Inf. Process. Syst..

[47] Simonyan K., Zisserman A. (2014). Very Deep Convolutional Networks for Large-Scale Image Recognition. arXiv.

[48] Xie S., Girshick R., Dollár P., Tu Z., He K. (2016). Aggregated Residual Transformations for Deep Neural Networks. arXiv.

[49] Zhang H., Wu C., Zhang Z., Zhu Y., Zhang Z., Lin H., Sun Y., He T., Muller J., Manmatha R. (2020). ResNeSt: Split-Attention Networks. arXiv.

[50] Howard A., Sandler M., Chu G., Chen L.C., Chen B., Tan M., Wang W., Zhu Y., Pang R., Vasudevan V. (2019). Searching for MobileNetV3. arXiv.

[51] Huang G., Liu Z., van der Maaten L., Weinberger K.Q. (2016). Densely Connected Convolutional Networks. arXiv.

[52] Ma N., Zhang X., Zheng H.T., Sun J. (2018). ShuffleNet V2: Practical Guidelines for Efficient CNN Architecture Design. arXiv.

[53] Tan M., Chen B., Pang R., Vasudevan V., Sandler M., Howard A., Le Q.V. (2018). MnasNet: Platform-Aware Neural Architecture Search for Mobile. arXiv.

[54] Redmon J., Farhadi A. (2018). YOLOv3: An Incremental Improvement. arXiv.

[55] Han K., Wang Y., Tian Q., Guo J., Xu C., Xu C. (2019). GhostNet: More Features from Cheap Operations. arXiv.

